# Association between Physical Fitness and Successful Aging in Taiwanese Older Adults

**DOI:** 10.1371/journal.pone.0150389

**Published:** 2016-03-10

**Authors:** Pay-Shin Lin, Chih-Chin Hsieh, Huey-Shinn Cheng, Tsai-Jou Tseng, Shin-Chang Su

**Affiliations:** 1 Department of Physical Therapy & Graduate Institute of Rehabilitation Science, College of Medicine, Chang Gung University, Taoyuan, Taiwan; 2 Health Aging Research Center, Chang Gung University & Chang Gung Memorial Hospital, Taoyuan, Taiwan; 3 Internal & Geriatric Medicine, Chang Gung Memorial Hospital, LinKou Branch, Taoyuan, Taiwan; Banner Alzheimer's Institute, UNITED STATES

## Abstract

Population aging is escalating in numerous countries worldwide; among them is Taiwan, which will soon become an aged society. Thus, aging successfully is an increasing concern. One of the factors for achieving successful aging (SA) is maintaining high physical function. The purpose of this study was to determine the physical fitness factors associated with SA in Taiwanese older adults (OAs), because these factors are intervenable. Community-dwelling OAs aged more than 65 years and residing in Northern Taiwan were recruited in this study. They received a comprehensive geriatric assessment, which includes sociodemographic data, health conditions and behaviors, activities of daily living (ADL) and instrumental ADL (IADL) function, cognitive and depressive status, and quality of life. Physical fitness tests included the grip strength (GS), 30-second sit-to-stand (30s STS), timed up-and-go (TUG), functional reach (FR), one-leg standing, chair sit-and-reach, and reaction time (drop ruler) tests as well as the 6-minute walk test (6MWT). SA status was defined as follows: complete independence in performing ADL and IADL, satisfactory cognitive status (Mini-Mental State Examination ≥ 24), no depression (Geriatric Depression Scale < 5), and favorable social function (SF subscale ≥ 80 in SF-36). Adjusted multiple logistic regression analyses were performed. Among the total recruited OAs (n = 378), 100 (26.5%) met the aforementioned SA criteria. After adjustment for sociodemographic characteristics and health condition and behaviors, some physical fitness tests, namely GS, 30s STS, 6MWT, TUG, and FR tests, were significantly associated with SA individually, but not in the multivariate model. Among the physical fitness variables tested, cardiopulmonary endurance, mobility, muscle strength, and balance were significantly associated with SA in Taiwanese OAs. Early detection of deterioration in the identified functions and corresponding intervention is essential to ensuring SA.

## Introduction

The aging crisis is spreading worldwide. Taiwan is no exception; its aging population is forecast to surpass 14% by 2017, and to rapidly increase to 20% by 2025 [1]. In this severe population aging era, if older people live longer but not in a healthy manner, caring for them will place an extreme burden on themselves, their families, and society. Therefore, successfully aging (SA) is a major health care and socioeconomic priority [2]. SA enables older people to lead an independent, high-quality, and dignified life. Rowe and Kahn [3] proposed the concept of SA and three constituent components: avoiding disease and disability, maintaining high cognitive and physical function, and engagement with life. A more comprehensive definition proposed by Young and colleagues [4] includes three health domains: physiological (e.g. diseases and functional impairments), psychological (e.g. emotional vitality), and social (e.g. spirituality and adaptation through social support mechanisms).

In the past four decades, scientist and clinicians have sought to develop a definition that would improve the general understanding of SA. In 2006, Depp and Jest [5] reviewed 29 studies, noticing several variations of the SA definition in the reviewed articles. They concluded that the most frequently described SA components were physical function or disability (26 of 29), followed by cognitive function, life satisfaction or well-being, and social or productive engagement.

Studies of different countries have proposed varying definitions of SA. In Spain, Formiga and colleagues [6] defined SA as achieving a Barthel Index (BI) scores of ≥90 and a Mini-Mental State Examination (MMSE) score of ≥24, as well as never having been institutionalized. In Singapore, Ng and colleagues [7] adapted the most rigorous criteria for defining SA. They defined SA as having good or excellent self-reported health status, being independent in performing instrumental activities of daily life (IADL), scoring ≥26 on the MMSE and ≤5 on the Geriatric Depression Scale (GDS), being engaged in at least one social and one productive activity, and reporting a high level of life satisfaction. In Australia, Parslow and colleagues [8] defined SA as reporting high-level physical and mental health and life satisfaction. Researchers have also varied in definitions concerning the three SA domains. Being independent in performing activities of daily life (ADL) or IADL has been frequently used for defining the physical component of SA. Several studies on SA conducted by MacArthur, investigating and longitudinally following OAs with high functioning, could be considered the earliest and most renowned studies on SA [9–11]. From then, many SA-related factors have been reported, such as health behaviors (smoking, physical activity, and exercise), comorbidity, nutrition, and some sociodemographic variables (age, sex, marital status, education, and living arrangement) [5–7,12–14].

High physical function is a crucial factor for SA; measures of functional performance and physical fitness are important, simple, and objective observations of physical function in older people. In addition, deterioration of these measures usually precedes functional dependence, which facilitates early detection and prevention. In the MacArthur studies, the maintaining of high physical performance was predicted by sociodemographic data and health status characteristics, exercise behavior, and emotional support from a social network [10,11]. Furthermore, several physical performance tests can detect the early onset of functional dependence in OAs without disabilities [15,16].

Recently, Kuh and colleagues [17] summarized the findings of a review on the indicators of healthy aging, focusing on objective measures of physical capability, such as grip strength, walking speed, chair rises, and standing balance. They concluded these standardized physical performance tests were significantly correlated with healthy aging. However, associations of these tests with SA needs to be further elaborated.

SA-associated factors may vary depending on cultural context. Few related studies have used Taiwanese older people as the study sample. In this study, we explored SA-associated factors and predictors, specifically physical fitness tests (PFTs), in Taiwan’s older population. Because physical fitness is trainable, early detection and intervention is possible.

## Methods

### Participants

Community-dwelling OAs aged more than 65 years were recruited through an internal medicine clinic and a community-based elderly home in Taoyuan, Taiwan. The subjects were screened by a medical doctor in the clinic and by two trained physical therapists in the elderly home. Subjects’ inclusion criteria were able to follow instructions and perform PFTs independently with or without assistive devices. The exclusion criteria were any health problems or acute trauma that would limit participation in the PFTs. The study was approved by the Institutional Review Board of Chang Gung Memorial Hospital, and written informed consent was obtained from each subject prior to the initial assessment.

### Measurements

#### Comprehensive geriatric assessment (CGA)

The subjects received a CGA by a well-trained research assistant. Data on sociodemographic characteristics were collected, including age, sex, marital status, educational level, and living arrangement. The health condition was assessed by recording the number of comorbidities and fall occurrences in the past year. Health behaviors were assessed including smoking and alcohol consumption (no, formerly, or yes), sleeping quality (good or insomnia), and physical activity or exercise (type, frequency, and duration). The level of independence was measured by ADL [18] and IADL [19]. The subjects were asked if any difficulty in performing six ADLs, namely eating, transferring, toileting, bathing, walking indoors, and dressing, and five IADLs, namely shopping, transportation, making phone calls, taking medications, and managing money. Cognitive function was measured using the Chinese version of the MMSE [20], which has been sufficiently validated in Taiwan [21,22]. Depression status was measured using the Chinese version of the GDS short form [23], which was adequately validated in Taiwan [24]. Quality of life was evaluated using the Chinese version of the Medical Outcomes study 36-Item Short Form (SF-36) [25]. The SF-36 comprises eight subscales with scores ranging from 0 to 100 for each scale and two weighted summary scales, namely the physical and mental component summary scores.

#### Physical fitness tests (PFTs)

PFTs consisted of the nine following assessment items. First, body composition was evaluated by calculating the body mass index (BMI).

Muscle strength was measured with the grip strength (GS) by using a Jamar® hand dynamometer (Sammons Preston, Bolingbrook, IL, USA) [26]. As recommended by the American Society of Hand Therapists, subjects were instructed to adopt the standard testing position [27], which is seated with shoulder and forearm of the test arm in a neutral position and the elbow flexed at 90°. The maximum force was recorded twice for the dominant hand and the higher value was used for analysis.

Muscle endurance was measured using the 30-second sit-to-stand (30s STS) test [28]. Subjects were instructed to keep their arms folded and rise as fast as possible from a seat, which was 0.42 m from the floor. The score was recorded as the number of full stands performed in 30 seconds.

Aerobic endurance was assessed using the 6-minute walk test (6MWT) [28]. Subjects were instructed to walk as fast and as far as possible along a 25-m corridor in 6 minutes and were given encouragement throughout the test. The total distance walked within 6 minutes was recorded.

Flexibility was assessed using the sit-and-reach test (SRT) [28]. Subjects completed trials by assuming a sitting position on the edge of a chair, with one leg extended and both hands overlapped and reaching toward the toes. The distance (in cm) from the extended third finger to the tip of the toe (+ beyond or–behind the toe) in two trials was recorded, and the better performance was used for analysis.

Balance was assessed using the functional reach (FR) test and one-leg standing (OLS) with eyes closed test. The FR test was performed using a Rolyan® Functional Reach Measuring Device (Sammons Preston). Subjects completed trials in a standing position adjacent to a wall with the test arm raised forward at 90° of shoulder flexion. The farthest distance (in cm) was recorded while subjects reached forward without taking their heels off the floor [29], and after practice, the mean value of two trials was used for analysis. In the OLS test, subjects completed trials by standing on one leg for as long as possible with the contralateral hip and knee both flexed at 90° and with their eyes closed [30]. The performance time was recorded until the suspended leg touched the floor, and after practice, the longest time of two trials was used for analysis.

The timed up-and-go (TUG) test [31] was used to assess dynamic balance and agility. Subjects stood up from a seated position, walked 3 m, turned around, and returned to the seated position on a chair. After a practice trial, the shortest time of two test trials was recorded.

Finally, the reaction time was assessed using the drop ruler (DR) test [32]. The subjects tried to catch a ruler that was dropped without warning. The distance (in cm) between the bottom of the ruler and the marking where the dropped ruler was caught by the subject was recorded. This procedure was repeated five times. The lowest and highest results were discarded and the average of the remaining three results was used for analysis.

### Definition of Successful Aging

Young [4] proposed a multidimensional model for SA that includes three domains, namely physiological, psychological, and sociological. Therefore, we operationally defined the criteria of SA status as respondents reporting follows:

1. Independence for performing each ADL and IADL activity [33,34].
2. Free of cognitive impairment and depressive symptoms if their MMSE score ≥24 [35] and GDS score <5 [24], respectively.
3. Having satisfactory social functioning if their social function subscale score of SF-36 was more than 80. We used 80 as a cutoff because in a normative sample of Taiwanese adults, 81 and 73 were the mean scores reported by healthy OAs aged 65–74 and >75 years, respectively [25].

According to these criteria, the subjects were categorized into two groups: SA and non-SA.

### Statistical Analysis

Descriptive statistics of sociodemographic characteristics, health conditions, health behavior, and PFTs were obtained for both the SA and non-SA groups. The *t* test was used for comparing continuous variables, and the chi-square test was used for categorical variables. To determine the variables associated with SA, series logistic regression analyses were performed. By using SA status as a dependent variable and possible correlated factors as independent variables, we constructed series logistic regression models. Sociodemographic characteristics, health conditions, and health behaviors were entered in model 1. For the univariate logistic regression (model 2), each PFT was added individually. Finally, all the variables of PFTs were included in a multivariate logistic regression (model 3).

Adjusted odds ratios (ORs) with 95% confidence intervals (CIs) were calculated. All analyses were performed using \SPSS 20.0. The significance level was set at *P* < 0.05.

Multiple imputation procedures were performed for missing values [36]. The procedure involved generating five complete datasets from a set of values yielded using the aforementioned logistic and linear regression models, for the categorical and continuous variables [37]. The results were similar after multiple imputations, therefore, results of the original data without imputations are reported in the present study.

## Results

This study enrolled 378 OAs with a mean age of 77.6 ± 6.9 years (60–102 years), 52.6% of whom were women. Regarding education, 55.2% of the subjects had received ≤ 9 years of education. Almost 60% of the subjects were married, and 70% lived with their families or others. Of the subjects, 202 (53.4%) were physically independent, 228 (60.3%) were cognitively and emotionally well-functioning, and 222 (58.7%) reported satisfactory social function. Overall, 100 (26.5%) subjects met the multidimensional criteria for SA (Table 1).

**Table 1:** Prevalence of successful aging.

N = 378	Prevalence
1. Good physical function	53.4
Independent of ADL	84.4
Independent of IADL	55.6
2. Good cognitive and emotional function	60.3
MMSE≧24	73.5
GDS<5	77.0
3. Well social function (score>80)	58.7
Successful aging^a^	26.5

Values are in %. ADL: activities of daily living; IADL: instrumental activities of daily living; MMSE: Mini-Mental State Examination; GDS: Geriatric Depression Scales.

^a^Combined criteria (1 and 2 and 3).

Table 2 shows the comparison results of the sociodemographic characteristics, health related factors, and physical fitness measures between SA and non-SA groups. Compared with the non-SA group, the subjects with SA were younger, had a higher level of education (>junior high school), and were more likely to live with others. They reported frequent regular exercise (≥2 days a week) and fewer falls (≤1 in the past year). Significant differences were observed in the height and weight of the SA group subjects, who were generally taller and heavier than the non-SA subjects; however, BMI did not differ significantly between two groups. In addition, no significant difference in the number of comorbidities was observed between two groups.

**Table 2:** Comparisons of measures of sociodemographic, health condition, health behavior and physical fitness tests between SA and non-SA.

		SA (n = 100)	Non-SA (n = 278)	*p* value
**Sociodemographic data**				
	Age (years)	75.91±7.31	78.20±6.70	0.004^a^
	Female, n(%)	47(47.0%)	152(54.7%)	0.187
	Education >9 years, n(%)	58(66.7%)	85(36.6%)	0.000^a^
	Married, n(%)	51(60.7%)	133(58.3%)	0.705
	Live with families or others, n(%)	50(59.5%)	167(73.2%)	0.019^a^
	Height (cm)	160.95±7.98	156.73±8.51	0.000^a^
	Weight (kg)	63.10±10.61	59.01±11.09	0.002^a^
	BMI	24.36±3.63	24.03±3.90	0.476
**Health condition**				
	Falls >1, n(%)	3(3.0%)	38(13.7%)	0.003^a^
	Comorbidity	1.24±1.00	1.41±1.05	0.181
**Health behavior**				
	Regular exercise, n(%)	88(88.0%)	181(65.6%)	0.000^a^
	No smoking, n(%)	82(82.8%)	218(79.3%)	0.446
	No drinking, n(%)	83(83.8%)	241(87.6%)	0.341
	Normal sleep, n(%)	73(73.7%)	182(66.9%)	0.210
**Physical fitness tests**				
	Grip strength (kg)	27.45±8.08	22.72±7.64	0.000^a^
	30s STS (no. stands)	15.53±5.36	12.68±4.86	0.000^a^
	6MWT (meter)	4.19±0.98	3.36±1.13	0.000^a^
	TUG (seconds)	9.86±2.83	14.45±8.47	0.000^a^
	Functional reach (cm)	28.53±5.83	23.62±6.69	0.000^a^
	One leg standing (seconds)	4.09±4.36	2.99±4.46	0.045^a^
	Chair sit-and-reach (cm)	0.45±13.16	-2.16±13.07	0.111
	Ruler test (cm)	35.59±9.29	39.68±9.61	0.000^a^

Values are in mean±SD unless otherwise stated. SA: successful aging; non-SA: no successful aging; BMI: body mass index; Regular exercise: having regular exercise at least two days a week for more than 20 minutes; Normal sleep: no insomnia; Falls>1: falls occurrence over once in the past year. 30s STS: 30-second sit to stand test; 6MWT: Six Minute Walk test; TUG: Timed Up and Go test.

^a^*p*<0.05

All PFTs, except the chair SRT, revealed significant differences between the SA and non-SA groups (Table 2). Compared with the non-SA group, the SA group achieved a stronger GS, performed more repetitions in the 30s STS test, walked a longer distance within 6 minutes, had a longer FR, required less time to complete the TUG test, caught the ruler higher in the ruler drop test, and stood for longer in the OLS test.

In the first logistic regression (model 1), age, education, and regular exercise were found to be significantly associated with SA; however, after multiple imputations, there was a minor change that the variable of height but not education showed significant difference (Table 3). In model 2, the GS, 30s STS, TUG, and FR tests as well as the 6MWT were significantly associated with SA after adjusting for sociodemographic characteristics, health conditions, and health behaviors (Table 4). Hosmer and Lemeshow tests revealed a good model fit for all models with no multicollinearity (none of the independent variables in the analyses had a standard error larger than 2.0), and an adequate overall accuracy rate (70.1%–75.5%). Significant PFT variables associated with SA and their unadjusted and adjusted ORs are presented in Table 4. For every 1-meter increase in the 6MWT, the odds of achieving SA were 2.057 times higher. By contrast, for every 1-second increase in the TUG test, the odds of achieving SA decreased by 19%.

**Table 3:** Factors associated with SA in model 1 from original and imputed data respectively.

Variables		OR (95% CI)	After multiple imputation OR (95% CI)
**Sociodemographic data**			
	Age	0.951 (0.907–0.999)^a^	0.956(0.918–0.996)^a^
	Gender	1.431 (0.543–3.773)	1.883(0.823–4.305)
	Education	2.375 (1.214–4.647)^a^	1.936(0.963–3.892)
	Marital status	1.384 (0.659–2.907)	1.115(0.570–2.179)
	Live arrangement	0.624 (0.290–1.340)	0.718(0.356–1.448)
	Height	1.062 (0.998–1.129)	1.084(1.027–1.143)^a^
	Weight	1.015 (0.982–1.049)	1.010(0.983–1.039)
**Health condition**			
	Falls >1	0.606 (0.124–2.953)	0.534(0.146–1.952)
	Comorbidity	1.112 (0.803–1.539)	0.964(0.728–1.277)
**Health behavior**			
	Regular exercise	2.414 (1.095–5.325)^a^	2.599(1.272–5.310)^a^
	No smoking	1.971 (0.748–5.194)	1.446(0.642–3.258)
	No drinking	0.792 (0.305–2.055)	0.861(0.378–1.961)
	Normal sleep	1.391 (0.704–2.748)	1.223(0.690–2.169)

SA: successful aging; OR: odds ratio; CI: confidence interval; BMI: body mass index; Regular exercise: having regular exercise for more than 20 minutes at least two days a week; Normal sleep: no insomnia; Falls>1: falls occurrence over once in the past year.

^a^*p*<0.05

**Table 4:** Physical fitness tests associated with SA in both unadjusted and adjusted univariate logistic regressions.

Physical fitness tests	Unadjusted	Adjusted
**Grip strength**	1.077(1.045–1.110) ^a^	1.077(1.018–1.139) ^a^
**30s STS**	1.116(1.062–1.173) ^a^	1.102(1.021–1.190) ^a^
**6MWT**	2.159(1.654–2.820) ^a^	2.057 (1.402–3.020) ^a^
**TUG**	0.795(0.728–0.869) ^a^	0.810 (0.708–0.927) ^a^
**Functional reach**	1.131(1.084–1.180) ^a^	1.089 (1.023–1.160) ^a^
**One leg standing**	1.053(0.995–1.116)	0.986 (0.919–1.058)
**Chair sit-and-reach**	1.015(0.996–1.035)	1.013 (0.984–1.043)
**Ruler test**	0.955(0.930–0.980) ^a^	0.972 (0.938–1.007)

Values are in odds ratio (95% confidence interval); Adjusted: adjusted for sociodemographic, health condition, health behavior measures.

30s STS: 30-second sit to stand test; 6MWT: Six Minute Walk test; TUG: Timed Up and Go test.

^a^*p*<0.05

However, after all the physical fitness variables were added to the multivariate logistic model, none were found to be significantly associated with SA (data not shown).

## Discussion

In our sample of 378 community-dwelling Taiwanese OAs, 26.5% were recognized as in a SA status. In addition to age, education, and regular exercise, we found some PFT variables, namely GS, 30s STS, 6MWT, TUG, and FR tests, to be significant factors associated with SA status.

Previous studies [5–7,12–14,33] have reported wide-ranging rates of SA prevalence, from 0.4% to 95%. Regarding other primarily ethic Chinese older populations, the SA prevalence in our study is similar to that reported in Singapore (28.6%) [7] but not to that reported in Hong Kong (0.7%–33.1%) [13] and in Shanghai (46.2%) [14]. Several methodological factors, such as differences in sample characteristics and operational definitions of SA, may contribute to this variability [5]. Self-reported and performance-based assessments are both valid and reliable methods for assessing ADL or IADL; however, in our study, using self-reported ADL or IADL measurements for defining SA may have led to overestimation of the number of subjects with SA. The heterogeneity and lack of consistency in the SA definition are the main limitations of research on SA [38]. However, we attempted to use the most suitable multidimensional definition of SA in our study.

The first logistic regression (model 1) revealed that some sociodemographic characteristics were associated with SA. In accordance with previous studies [13,14], our results showed that a younger age and higher level of education were associated with SA. However, the number of comorbidities was not a significant factor. Previous studies [4,8,39] have also proposed that chronic illness is not a necessary barrier to SA. By contrast, performing regular exercise for ≥20 min more than twice a week is beneficial. According to one study [40], the SA rate in the physically active Canadian older population was more than twice that in older populations of other countries, even after adjusting for demographic covariates. Maintaining a high physical activity level by performing regular exercise results in improved physical function and fitness, and thus aids older people to achieve SA.

Our results showed that GS, 30s STS, 6MWT, TUG, and FR test results were significant factors associated with an SA status. A review performed by den Ouden and colleagues [41] describes positive associations of high handgrip and lower extremity strength and quick gait speed on physical mobility. Results of a 10-year follow-up study [42] also reported that high handgrip strength and leg strength were both associated with a lower risk of ADL disabilities.

Formiga and colleagues [6] conducted a survey involving 328 community-dwelling OAs. Results showed that successful agers exhibited significantly higher scores on the Tinetti Gait Scale than did unsuccessful agers. Achour and colleagues [12] used a physical questionnaire to evaluate the amount of physical activity in 686 subjects aged over 65 years and determined the relationships between exercise capacity and life satisfaction and self-rated health after 7 years. The results showed oxygen uptake (VO_2_) peak and activity index were the most significant factors associated with self-reported health condition and life satisfaction. Our results were in agreement with those of the aforementioned studies. In conclusion, older people with more favorable muscle strength/endurance, gait and balance performance, and aerobic endurance exhibit higher physical function, exercise capacity, and independence in performing ADL; thus, they are more able to achieve SA.

PFTs are easy to conduct in clinical and community settings. Previous research showed their predictability on adverse outcomes. Some tests, such as GS, timed chair raises, walking speed, and standing balance, can predict mortality [43]; tests such as timed chair raises [44] and TUG [45] can predict disability, and others such as gait speed can predict demand for personal care [46]. Rikli and Jones [47] developed standards for tests that can predict the level of capacity required for OAs to maintain physical independence, which is a necessary component of SA. For example, to predict a high probability of maintaining independence, the recommended score for the 30s STS test is ≥14 for women aged 70–74 years. Thus, an appropriate exercise regimen can be prescribed by health professionals aimed toward improving lower extremity muscle strength and endurance to achieve satisfactory performance in the 30s STS test, i.e. physical function improvement. Therefore, PFTs are valuable for OAs to monitor their functional status, and for professionals to screen and detect OAs at high risk.

In this study, some PFTs, such as OLS, chair SRT, and DR tests showed no significant association with SA. Data of the OLS test was not normally distributed and exhibited the floor effect. Therefore, more functional and dynamic balance tests, such as TUG and FR, could be more suitable options than the OLS for measuring the balance performance in the older population.

Flexibility, a variable closely relevant to pain and discomfort, is also necessary for OAs to maintain a better functional state and remain independent. A similar result to our study was reported by Chow et al [48]; they investigated the association of out-of-home activities and physical fitness with SA, and found that muscular strength and cardiovascular fitness were significant factors for SA, but not flexibility. Limited joint range-of-motion is more closely associated with functional state than is flexibility. Unsatisfactory flexibility may not represent a joint range-of-motion limitation; however, it influences body functioning and dependence ability. The mean of chair SRT tests in our study were similar to those reported in a previous study [49].

The DR test represents the reaction time. Our results showed that a quick reaction time or agility is not a crucial determinant of SA.

The final multivariate logistic model found that no PFT variables were significantly associated with SA after adjustment. After excluding multicollinearity, it is possible that the sample size in our study was insufficient and may have caused model instability.

In addition to an insufficient sample size, this study has several other limitations. First, the study was based on a convenience sample from Northern Taiwan. Different regions may possess different SA-associated factors; therefore, the results should be generalized with caution, and further research should include random sampling of a representative geographical areas. Moreover, we did not include OAs who could not perform the PFTs. Although they did not meet the SA criteria described in our study, this does not mean that they were unsuccessful agers. Montross et al [39] reported that of subjects who rated themselves as successful agers, only 30% were free of disability. SA can be considered a subjective concept; however, in this study, we attempted to define it objectively. SA is a multidimentinal concept, which can be achieved through adaption and compensation [4]. Thus, our results cannot be generalized to all the older population.

Second, the social domain of the SA definition adopted in our study may not be a standard measure. The SF-36 score may not fully represent the extent of a person’s engagement with life. A measurement bias may have developed. Third, certain data were missing in our study; however, the results were similar after multiple imputation procedures.

Finally, we conceptually defined independence in performing ADL or IADL as the physical component of SA, which logically may be correlated with improved physical fitness. However, the measures of physical fitness representing mobility function is in a different construct to the ADL measure. Improved physical fitness can help to achieve independence in performing ADL; however, a successful ager who is completely independent in performing ADL or IADL may not necessarily have strong physical fitness. Moreover, deterioration of the SA-related fitness measures usually precedes functional dependence, which makes early detection and prevention possible.

## Conclusions

In this study, we aimed to determine the factors associated with SA among Taiwanese community-dwelling OAs. In the sample of 378 subjects, 26.5% of them were successful agers. Our results suggested that, apart from the known factors of age, education level, and regular exercise, PFTs are also SA-associated significant factors. Favorable cardiopulmonary endurance, muscle strength and endurance, and balance and mobility are crucial SA-associated factors. However, these factors could be cause or effect. A longitudinal study is required to investigate the causality of the determined factors. PFTs are easy to perform in community or clinical settings. Thus, these tests are recommended for the early detection and monitoring of SA status in OAs.
